# The Impact of Disease-Related Fear and Internalized Stigma on Quality of Life in Patients with Scabies: A Cross-Sectional Study

**DOI:** 10.3390/healthcare14111575

**Published:** 2026-06-04

**Authors:** Nurperihan Tosun, Mustafa Tosun, Sermed Doğan, Mustafa Younis

**Affiliations:** 1 Department of Health Management, Faculty of Health Sciences, Sivas Cumhuriyet University, Sivas 58140, Türkiye; nurperihankarabulut@gmail.com; 2 Department of Dermatology, Faculty of Medicine, Sivas Cumhuriyet University, Sivas 58140, Türkiye; 3 Department of Health Institutions Management, Vocational School of Social Sciences, Kayseri University, Kayseri 38030, Türkiye; sermeddogan@kayseri.edu.tr; 4 Department of Health Policy and Management, Jackson State University, Jackson, MS 39217, USA; j00103323@jsums.edu

**Keywords:** scabies, internalized stigma, fear of disease, quality of life

## Abstract

Background/Objectives: Scabies is a contagious dermatological infestation that can cause not only physical symptoms but also considerable psychosocial burden. This study aimed to investigate the relationships between fear of scabies, internalized stigma, and dermatology-related quality of life in patients with scabies. Methods: This cross-sectional study included 131 patients diagnosed with scabies in a dermatology outpatient clinic. Data were collected using a structured questionnaire including sociodemographic and clinical characteristics, the Fear of Scabies Scale (FSS), the Internalized Stigma Scale (ISS), and the Dermatology Life Quality Index (DLQI). Correlation and regression analyses were conducted to examine the associations between fear of scabies, internalized stigma, and quality of life. Results: The mean DLQI score was 15.82 ± 5.69, indicating a considerable impairment in dermatology-related quality of life. Fear of scabies showed a weak but significant positive correlation with DLQI scores (r = 0.326, *p* < 0.001), whereas internalized stigma demonstrated a stronger correlation (r = 0.484, *p* < 0.001). Among the stigma subdimensions, social withdrawal showed the strongest association with impaired quality of life (r = 0.622, *p* < 0.001). Regression analyses revealed that internalized stigma explained 23% of the variance in DLQI scores (R^2^ = 0.234), while fear of scabies explained 10% (R^2^ = 0.106). In addition, longer symptom duration (β = 0.708, *p* < 0.001), nocturnal pruritus (β = 0.408, *p* = 0.009), and visible skin lesions (β = 0.263, *p* = 0.002) were associated with higher levels of fear of scabies. Conclusions: Internalized stigma and disease-related fear were associated with reduced quality of life, with stigma-related mechanisms appearing to play a particularly prominent role. These findings suggest that addressing stigma and providing psychosocial support may be important components of comprehensive scabies management.

## 1. Introduction

Scabies is a highly contagious skin infestation caused by the ectoparasite *Sarcoptes scabiei* var. hominis. It is characterized by intense itching and is primarily transmitted through prolonged skin-to-skin contact [1]. It is considered not only a clinical skin disease but also a significant global public health burden, particularly in developing countries, due to its prevalence as one of the most common dermatological diseases, its potential for spread associated with crowded living conditions, and its ability to predispose individuals to secondary bacterial infections [2,3]. Scabies is recognized by the World Health Organization (WHO) as a neglected tropical disease and affects approximately 200 million people worldwide at any given time [4,5].

Recent studies have reported increasing scabies incidence in several European countries, including Italy, Greece, Spain, and Turkey, particularly following the COVID-19 pandemic [6,7,8,9,10]. In Turkey, this increase has shown an epidemic-like trend during and after the pandemic period [10,11,12].

The clinical presentation of scabies is most commonly characterized by intense itching, particularly at night. The tunnels created by the mite within the epidermis and the resulting immune response lead to papulovesicular lesions in typical areas of infestation; intense scratching can compromise skin integrity, predisposing the skin to secondary bacterial infections [13]. In addition to these physical symptoms, the infectious nature of the disease and visible skin lesions can also cause psychological distress in individuals [14]. It is thought that this psychological impact, combined with societal perceptions linking the disease to hygiene and contagion, may pave the way for the development of internalized stigma in individuals. It is argued that the developing perception of stigma may negatively affect diagnosis and treatment processes by leading to concealment of the disease and delays in seeking healthcare services [15,16]. Indeed, studies have reported that in skin diseases, the feeling of stigmatization can increase the perception of social rejection, leading individuals to avoid disclosing their illness and develop social withdrawal behavior; this can complicate the management of the disease [17,18]. In addition, the infectious nature of the disease, concerns about transmitting it to others, misinformation about the disease, negative social perceptions, and the fear of reinfection or the disease being uncontrollable can cause individuals to develop a pronounced fear of the disease [19,20]. Increased fear related to the disease may trigger social avoidance and shame-related behaviors, thereby deepening the psychosocial burden. It may also contribute to social isolation by increasing perceptions of discrimination and feelings of exclusion. It is thought that over time, this process can become a difficult psychological burden for patients and negatively affect their quality of life [15,21]. Scabies is considered a multidimensional dermatological infestation associated with substantial morbidity due to both its clinical symptoms and its emotional and psychosocial effects [22].

Studies conducted using the Dermatology Life Quality Index (DLQI) [23], which is widely used in assessing quality of life in dermatological diseases, show that factors such as psychological impact, changes in physical appearance, and social stigma significantly affect quality of life in relation to lesion size, affected body areas, and symptom duration [24,25,26,27].

This study aimed to evaluate internalized stigma and disease-related fear in patients with scabies and to investigate their relationship with quality of life. We hypothesized that disease-related fear and internalized stigma would be associated with impaired quality of life.

## 2. Materials and Methods

### 2.1. Ethics

This cross-sectional study was conducted in the dermatology outpatient clinic of Sivas Cumhuriyet University Faculty of Medicine. Patients diagnosed with scabies during routine dermatological examination and who agreed to participate were consecutively included in the study. The Cumhuriyet University Ethics Committee approved our study (approval number: 668028, date: 23 January 2026). Sample size calculation was performed using G*Power software, version 3.1.9.7 (Heinrich Heine University, Düsseldorf, Germany). According to the point-biserial correlation model (effect size = 0.30, α = 0.05, power = 0.99), the minimum required sample size was calculated as 111 participants. In total, 131 patients with scabies were included in the study, exceeding the required sample size. The study was conducted in accordance with the principles of the Helsinki Declaration, the Patient Rights Regulation, and accepted ethical standards. All participants were provided with detailed information about the study procedures, and written informed consent was obtained. Patients with a psychiatric comorbidity, cognitive impairment, or who were unable to complete the questionnaires were excluded from the study in order to minimize potential confounding factors that could independently affect psychological outcomes and quality of life.

The sociodemographic characteristics of the patients (age, gender, marital status, education level, smoking habits, and alcohol consumption) were documented. In addition, clinical characteristics related to scabies, such as duration of symptoms, lesion localization, and treatment history, were documented, taking into account factors that could affect quality of life and psychological responses. Quality of life was assessed using the DLQI, one of the most commonly used measurement tools for evaluating quality of life in dermatological diseases; fear related to the disease was assessed using the Fear Scale for Scabies (FSS), and internalized stigma was assessed using the Internalized Stigma Scale (ISS).

### 2.2. The Dermatology Life Quality Index

The DLQI is a quality of life scale designed specifically for skin diseases and is widely used. It consists of 10 questions covering symptoms and feelings, daily activities, leisure time, work and school, personal relationships, and treatment. The scale items are answered by participants as “not at all”, “a little”, “a lot”, and “very much”. Accordingly, each item is scored between 0 and 3, and the total score ranges from 0 (no impact on quality of life) to 30 (very large impact on quality of life). A higher total score indicates a greater impact of the disease on the six different domains assessed by the scale [28].

In the literature, the reliability of the scale has been reported previously [29,30,31]. The Turkish version of the DLQI has been shown to be a valid and reliable instrument for use among dermatologists and dermatology patients. The internal consistency coefficient of the scale (α = 0.85) was found to be quite satisfactory [28]. In the present study, the reliability coefficient of the scale was calculated as 0.84.

### 2.3. Fear of Scabies Scale

Studies focusing specifically on scabies and raising awareness for its prevention and control are extremely limited in the literature. The Fear of Scabies Scale (FSS) was used to assess the level of fear or discomfort associated with the disease. The scale was originally developed and subsequently applied in studies evaluating fear related to scabies. The items of the scale (FSS-10) are rated on a four-point Likert scale as “strongly agree”, “agree”, “disagree”, and “strongly disagree”. The FSS consists of 10 items, and the possible total scores obtained from the responses range from 10 (lower level of fear) to 40 (higher level of fear). Scores below 33 (<33) indicate a lower level of fear, whereas scores ≥ 33 indicate a higher level of fear [19,20].

The Cronbach’s alpha coefficient of the scale was reported as 0.73 by Singg (2016) [19]. The Turkish validity and reliability study of the scale used in this research was conducted by Sofuoglu et al. (2023), who reported a Cronbach’s alpha coefficient of 0.69 [32]. In the present study, the reliability coefficient of the scale was calculated as 0.78.

### 2.4. Internalized Stigma Scale

The Internalized Stigma Scale (ISS) was originally developed as the Internalized Stigma of Mental Illness (ISMI) scale to measure subjective stigma, such as feelings of alienation and discrimination, among individuals with mental illness. In subsequent years, the scale has been used not only for psychological disorders but also in various other conditions and contexts in the literature. The ISS consists of five subscales: alienation (6 items), stereotype endorsement (7 items), perceived discrimination (5 items), social withdrawal (6 items), and stigma resistance (5 items), comprising a total of 29 items. Each item is rated on a 4-point Likert scale ranging from 1 (strongly disagree) to 4 (strongly agree). The total score obtained from the ISS ranges from 29 to 116, with higher scores indicating a greater level of internalized stigma. An internal consistency reliability coefficient of 0.90 has been reported for the scale. For the subscales, the reliability coefficients were reported as 0.79 for alienation, 0.72 for stereotype endorsement, 0.75 for perceived discrimination, 0.80 for social withdrawal, and 0.58 for stigma resistance [33].

The Turkish validity and reliability study of the scale has been conducted previously. The researchers reported Cronbach’s alpha coefficients ranging from 0.63 to 0.84 for the subscales, which were higher than those obtained in the original English version [34]. In the present study, the overall reliability coefficient of the scale was found to be 0.94. According to the subscales, the reliability coefficients were 0.75 for alienation, 0.81 for stereotype endorsement, 0.83 for perceived discrimination, 0.87 for social withdrawal, and 0.51 for stigma resistance. Although the subscale for stigma resistance falls below the accepted cutoff value, the subscale is frequently reported in the literature to have low internal consistency scores [33,35].

### 2.5. Statistical Analysis

All statistical analyses were performed using IBM SPSS Statistics for Windows Version 23.0 (IBM Corp., Armonk, NY, USA). The normality of the variables was evaluated using skewness and kurtosis values together with the Shapiro–Wilk test, and the distribution was considered acceptable when skewness and kurtosis values were within ±2. Descriptive statistics were presented as mean ± standard deviation, minimum–maximum values, frequencies, and percentages.

The internal consistency reliability of the scales was assessed using Cronbach’s alpha coefficients. Relationships between variables were evaluated using Pearson correlation analysis. In order to examine the relationship between fear of scabies and other variables, simple and multiple linear regression analyses were performed. Prior to the regression analysis, the assumptions were found to hold after examining linearity, multicollinearity, and the independence and normality of the error terms. Categorical variables were coded as dummy variables prior to the regression analysis (e.g., gender: male = 0, female = 1; marital status: married = 0, single = 1). The reference category was excluded from the model to avoid multicollinearity (dummy variable trap). A *p*-value < 0.05 was considered statistically significant.

## 3. Results

### 3.1. Demographic and Clinical Characteristics

The demographic and clinical characteristics of patients with scabies and the differences related to fear of scabies are presented in Table 1. The mean age of the patients was 35.87 ± 15.58 years. In terms of gender distribution, the proportion of male participants (64.1%) was higher than that of females (35.9%). More than half of the participants (51.9%) were married. Regarding educational status, the majority of the participants (60.3%) had a high school education. Similarly, in terms of lifestyle habits, most participants did not consume alcohol (84.7%) or smoke (58.0%).

Among the patients, 95.4% were living at home, while 4.6% were living in dormitories. A total of 40 patients (30.5%) reported that there were other individuals with scabies in their living environment. The mean duration of symptoms was 80.53 ± 76.75 days. Lesions were most frequently reported in the genital region (16.1%), followed by the wrist (12.5%), chest (10.7%), legs (10.5%), abdomen (8.9%), forearm (8.0%), gluteal region (5.6%), inguinal region (5.6%), arm (3.9%), axillary area (3.9%), and interdigital spaces (2.5%). It is observed that the highest number of combinations occurs in the genital, wrist, and chest areas (26.7%). Regarding skin lesions, excoriations (74.2%), papules (22.0%), and secondary infections (3.8%) were reported among the participants. Participants were grouped according to the predominant type of skin lesion.

In terms of treatment, 20.7% of the patients reported that they had not received any treatment, while 56.6% had received treatment once, 10.3% twice, and 12.4% three or more times. The treatments most frequently used were permethrin cream (81.4%) and precipitated sulfur (79.8%). Following treatment, 73.2% of the patients reported a positive response to treatment, whereas 26.8% stated that they did not respond to treatment. Finally, 62.6% of the participants reported that they had no additional diseases, and 67.9% reported that they were not using any other medications.

The responses given by the patients to the scale items were analyzed and mean scores were calculated. Accordingly, the mean score obtained from the FSS was 29.68 ± 5.82. According to the scoring system of the scale, scores below 33 indicate a lower level of fear. Therefore, based on the average scores of the participants, the overall level of fear related to scabies was relatively low.

The mean score of the ISS was 58.61 ± 14.58, which is slightly above the midpoint of the scale range. This finding suggests that participants with scabies experienced a moderate level of internalized stigma.

The mean score of the DLQI was 15.82 ± 5.69. Considering that the maximum score of the scale is 30, this result indicates that scabies had a considerable negative impact on the quality of life of the participants.

### 3.2. Findings Regarding the Relationship Between Variables

Pearson correlation analysis was conducted to examine the relationships between the study variables. The results indicated that the FSS showed a weak but statistically significant positive correlation with the DLQI (r = 0.326, *p* < 0.01). However, no statistically significant relationship was found between FSS and the ISS (r = 0.069, *p* > 0.05). Similarly, no significant correlations were observed between FSS and the subdimensions of the ISS.

A moderate positive and statistically significant correlation was found between DLQI and ISS (r = 0.484, *p* < 0.01). In addition, DLQI demonstrated moderate positive correlations with the alienation (r = 0.527, *p* < 0.01) and perceived discrimination (r = 0.574, *p* < 0.01) subscales of the ISS. A strong positive correlation was observed between DLQI and the social withdrawal subscale (r = 0.622, *p* < 0.01). Furthermore, DLQI showed a weak but significant positive correlation with the stereotype endorsement subscale (r = 0.255, *p* < 0.01). The Type I error rate was taken into account in the context of multiple correlation analysis. In this regard, a Benjamini–Hochberg False Discovery Rate (FDR) correction was applied, and it was determined that the correlations found to be statistically significant retained their significance. The correlation results among the variables are presented in Table 2.

### 3.3. Findings Related to Linear Regression Analyses

The relationships between FSS, ISS, and DLQI were examined using simple linear regression analysis. According to the results, a positive relationship was observed between FSS and DLQI (β = 0.326). FSS was significantly associated with DLQI (*p* < 0.001), and FSS explained approximately 10% of the variance in DLQI.

Similarly, a positive relationship was found between ISS and DLQI (β = 0.484). ISS was significantly associated with DLQI (*p* < 0.001), explaining approximately 23% of the variance in DLQI. Therefore, the regression models examining the associations of FSS and ISS on DLQI were found to be statistically significant.

In addition, according to the results of the multiple linear regression analysis, both FSS (β = 0.294) and ISS (β = 0.464) were significantly and positively associated with DLQI. These findings indicate that fear of the disease and internalized stigma together show a stronger association with quality of life. The results of the analyses are presented in Table 3.

Results of the regression analysis examining the associations between ISS subdimensions on DLQI are presented in Table 4. According to the findings, the ISS subdimensions of alienation (β = 0.527), stereotype endorsement (β = 0.255), perceived discrimination (β = 0.574), and social withdrawal (β = 0.622) demonstrated significant positive associations with DLQI. Among these dimensions, social withdrawal showed the strongest association with quality of life impairment.

In terms of explained variance, alienation accounted for 22% of the variance in DLQI, stereotype endorsement explained 6%, perceived discrimination explained 33%, and social withdrawal explained 38% of the variance. In contrast, the stigma resistance subdimension was not significantly associated with DLQI.

The results regarding the demographic (age, gender, marital status, education level, and place of residence) and clinical characteristics (duration of symptoms, nocturnal pruritus, skin lesions, and regional involvement) of the patients are presented in Table 5. According to the linear regression analysis, gender was significantly associated with fear of scabies (β = −0.224, *p* < 0.05). This finding indicates that men’s levels of fear of scabies are lower than those of female participants. In contrast, education level (β = 0.468, *p* < 0.001), duration of symptoms (β = 0.708, *p* < 0.001), nocturnal pruritus (β = 0.408, *p* < 0.05), skin lesions (β = 0.263, *p* < 0.05), and regional involvement (β = 0.073, *p* < 0.05) were positively associated with fear of scabies. Age, marital status, and place of residence were not significantly associated with fear of scabies.

The results of the multiple regression analysis of the DLQI in relation to other clinical variables are presented in Table 6. According to the analysis results, among the variables included in the model, only the treatment response was significantly associated with quality of life (β = −0.708, *p* < 0.001). This finding indicates that individuals who responded positively to treatment had lower DLQI scores. No significant associations were observed between the other variables and quality of life.

## 4. Discussion

Scabies is increasingly recognized not only as a dermatological infestation but also as a disease associated with significant psychosocial consequences. In this study, we examined the relationships between disease-related fear, internalized stigma, and dermatology-specific quality of life in patients with scabies. Our findings indicate that both fear of scabies and internalized stigma are significantly associated with impaired quality of life. These results demonstrate that the burden of scabies is not limited to physical symptoms but also encompasses important psychological and social dimensions [16,36].

The mean DLQI score obtained in our study indicates a substantial deterioration in the quality of life of patients with scabies. The DLQI levels observed were consistent with findings from previous studies conducted in Brazil, Ethiopia, and the Solomon Islands, all of which demonstrated marked impairment in dermatology-related quality of life among individuals with scabies [21,22,25]. Persistent pruritus, sleep disturbances, visible skin lesions, and fear of contagion may negatively affect daily activities, occupational performance, and interpersonal relationships, leading to social withdrawal and reduced productivity. In addition, treatment costs and work absenteeism may further increase psychosocial burden and impair overall well-being [37]. These findings are in line with previous studies showing that scabies significantly affects emotional well-being, social functioning, and health-related quality of life through both physical symptoms and psychosocial stressors [12,21,25].

Correlation analyses have shown that both fear of scabies and internalized stigma are significantly associated with dermatology-specific quality of life. However, the strength of these relationships varies. Internalized stigma showed a stronger relationship with DLQI scores, while fear related to the disease showed a weaker relationship. This suggests that psychosocial mechanisms related to stigma may show a stronger association with patients’ well-being than disease-related fear [16]. However, these associations should be interpreted with caution, as not all potential clinical confounding variables were simultaneously included in the regression models. Therefore, the observed associations do not necessarily indicate independent relationships between fear of scabies, internalized stigma, and dermatology-specific quality of life.

Regression analyses further supported these findings. Internalized stigma demonstrated a stronger association with impaired quality of life, whereas fear of scabies showed a comparatively lower explanatory contribution. When both variables are included in the model together, they explain a larger portion of the variance in quality of life. These findings suggest that psychosocial processes, particularly internalized stigma, may play an important role in the burden associated with scabies.

This study also demonstrates that internalized stigma is a multidimensional construct that affects quality of life in various ways. Among the subdimensions of stigma, social withdrawal showed the strongest correlation with DLQI scores. Patients who avoid social interactions due to shame, fear of judgment, or concern about transmitting their illness to others may receive less social support and experience greater psychological distress. It has been previously reported that social avoidance due to stigma in dermatological diseases significantly impairs quality of life [16,18].

In addition to social withdrawal, alienation and perceived discrimination have also shown a significant relationship with deterioration in quality of life. Alienation refers to the individual feeling different from others or socially isolated due to their illness. In the context of scabies, visible skin lesions and the contagious nature of the disease can lead patients to feel socially excluded. Similarly, perceived discrimination refers to an individual’s belief that they will be treated negatively by others due to their illness, which can increase psychological distress. Studies in dermatology also show that perceived discrimination in visible skin diseases is an important mechanism in the decline of quality of life [38,39].

The stereotype confirmation dimension has also been found to be related to quality of life, but the strength of this relationship is lower than that of other dimensions. This suggests that some patients may internalize the societal misconception that scabies is associated with poor hygiene or adverse living conditions. Internalizing such stereotypes can lead to feelings of shame and self-blame, negatively affecting psychological well-being [36].

Interestingly, despite the relatively low overall level of scabies fear in our sample according to the Scabies Fear Scale scoring criteria, the deterioration in quality of life is quite pronounced. This suggests that psychosocial factors such as stigmatization and social reactions may play a greater role than disease fear in determining patients’ well-being. Certain clinical factors have also been found to be associated with increased fear of scabies. In particular, prolonged symptom duration showed the strongest association with fear levels. Persistent itching and visible lesions may cause patients to experience uncertainty about recovery and increased anxiety levels. Similarly, nocturnal pruritus and visible skin lesions have also been found to be associated with fear levels.

It has been previously reported that prolonged symptoms in dermatological diseases increase psychological burden and reduce quality of life [26]. The finding that educational level is positively correlated with fear suggests that individuals with higher educational levels may be more aware of the contagiousness of the disease and its social consequences.

From a public health perspective, these findings are quite significant given the global burden of scabies. Classified by the World Health Organization as a neglected tropical disease, scabies affects more than 200 million people worldwide at any given time. Beyond its dermatological symptoms, the disease can lead to significant psychosocial consequences such as stigmatization, social exclusion, and psychological distress. These psychosocial effects may be associated with an increased overall burden of the disease [5,36].

From a clinical perspective, these findings indicate that psychosocial aspects should also be considered in the management of scabies. The recent increase in scabies incidence reported in several countries following the COVID-19 pandemic may be associated with disruptions in healthcare access, increased household transmission, and treatment-related challenges. In addition, “pseudoresistance,” which may result from inadequate treatment duration, poor adherence, or incorrect application of therapy, has been suggested as a possible contributing factor to the persistence and spread of the disease [6,12]. These factors may also contribute to increased psychosocial burden, disease-related fear, and concerns about contagion among affected individuals. Dermatologists and other healthcare professionals should be aware that patients may experience stigmatization, shame, and social withdrawal due to the disease. Therefore, providing appropriate counseling to patients, emphasizing that scabies is a treatable disease, and providing accurate information about disease transmission can play an important role in reducing psychological distress and improving patient outcomes.

Overall, the findings of this study suggest that scabies should be considered not only as a parasitic skin infestation but also as a disease with significant psychological and social consequences. Therefore, effective management strategies should not be limited to pharmacological treatment alone but should also include approaches such as patient education, reduction in stigma, and psychosocial support.

One strength of the present study is that it simultaneously evaluates disease-related fear and internalized stigma in relation to dermatology-specific quality of life in patients with scabies, which has rarely been addressed in the previous literature.

Our study has several limitations. First, its cross-sectional design does not allow causal inferences regarding the relationships between disease-related fear, internalized stigma, and quality of life. Second, the study was conducted at a single center with a relatively modest sample size, which may limit the generalizability of the findings to other populations and healthcare settings. The predominance of male participants may also have influenced the representativeness of the psychosocial findings. Third, psychological variables were assessed using self-report questionnaires, which may be subject to response bias. Although the ISS has been used in various medical conditions, it was originally developed for psychiatric populations, which may limit its construct validity in dermatological conditions such as scabies. In addition, the stigma resistance subdimension demonstrated relatively low internal consistency, and findings related to this subscale should therefore be interpreted cautiously. Although individuals with known psychiatric disorders were excluded, other psychological factors such as anxiety and stress were not directly evaluated. Furthermore, not all potential clinical confounding variables were simultaneously included in the regression models evaluating DLQI. Another limitation is that no validated objective scabies severity scoring system was used, limiting interpretation of the relationship between clinical disease burden, psychological distress, and quality of life. Information regarding reasons for not receiving treatment or whether patients had received adequate treatment and hygiene instructions was also not systematically assessed. Finally, symptom duration was based on patient self-report and may partly reflect persistent post-scabetic pruritus rather than ongoing active infestation in some patients. Multicenter longitudinal studies are needed to better clarify the psychosocial burden of scabies and to develop interventions aimed at improving patient well-being.

## 5. Conclusions

The results of our study suggest that scabies is associated with significant psychosocial consequences beyond its dermatological manifestations. Fear related to the disease and internalized stigma were identified as important factors associated with impaired dermatology-specific quality of life. However, these findings should be interpreted with caution given the cross-sectional design of the study and the fact that not all potential clinical confounding variables were simultaneously included in the regression models. Mechanisms related to stigma, particularly social withdrawal, play a significant role in shaping patients’ well-being.

Although the overall level of fear of scabies is relatively low, the disease has a marked negative impact on quality of life. This finding suggests that psychosocial factors such as stigma and social reactions are closely associated with the overall burden of disease.

These findings highlight the need for a comprehensive approach to scabies management that includes not only pharmacological treatment but also patient education, reduction in stigma, and psychosocial support. Larger, multicenter studies will contribute to a better understanding of the psychosocial effects of scabies and the development of patient-centered management strategies.

## Figures and Tables

**Table 1:** Examination of the sociodemographic and clinical characteristics of scabies patients and their disease fear scores.

Variables	*n* (%) or Mean ± SD (Min–Max) (*n* = 131)	Median FSS	*p*
Age	35.87 ± 15.58 (20–72)		
Gender			0.039
Female	47 (35.9%)	34.0	
Male	84 (64.1%)	29.0	
Working status			0.001
No	68 (51.9%)	33.0	
Yes	63 (48.1%)	28.0	
Marital status			0.560
Single	63 (48.1%)	30.0	
Married	68 (51.9%)	30.5	
Education level			0.010
Primary education	6 (4.6%)	-	
Secondary education	20 (15.3%)	33.0	
High school	79 (60.3%)	30.0	
Higher education	26 (19.8%)	27.0	
Smoking status			0.403
Smoker	55 (42.0%)	32.0	
Non-smoker	76 (58.0%)	30.0	
Alcohol status			0.005
Drinking	20 (15.3%)	33.0	
does not drink	111 (84.7%)	30.0	
Place of residence			0.021
Home	125 (95.4%)	30.0	
Dormitory	6 (4.6%)	-	
Are there any other patients with scabies?			0.154
No	91 (69.5%)	31.0	
Yes	40 (30.5%)	29.0	
Duration of symptoms	80.53 ± 76.75 (20–365)		
FSS	29.68 ± 5.82 (12–40)		
ISS	58.61 ± 14.58 (38–102)		
DLQI	15.82 ± 5.69 (7–26)		

Abbreviations: FSS, Fear of Scabies Scale; ISS, Internalized Stigma Scale; DLQI, Dermatology Life Quality Index; SD, Standard Deviation.

**Table 2:** Arithmetic mean, standard deviation, and correlation results for variables.

	Mean	SD	1	2	3	4	5	6	7	8
FSS	2.79	0.58	(0.78)							
DLQI	1.44	0.52	0.326	(0.84)						
ISS	2.02	0.50	0.069	0.484	(0.94)					
Alienation	2.06	0.57	0.098	0.527	0.966	(0.75)				
Stereotype endorsement	1.94	0.58	0.126	0.255	0.894	0.857	(0.81)			
Perceived discrimination	1.87	0.69	−0.100	0.574	0.872	0.823	0.670	(0.83)		
Social withdrawal	1.94	0.59	0.160	0.622	0.928	0.932	0.780	0.826	(0.87)	
Stigma resistance	2.34	0.52	−0.024	0.021	0.536	0.416	0.389	0.323	0.316	(0.51)

Abbreviations: FSS, Fear of Scabies Scale; DLQI, Dermatology Life Quality Index; ISS, Internalized Stigma Scale; SD, Standard Deviation.

**Table 3:** Associations of fear of scabies and internalized stigma with quality of life.

		Non-Standardized Coefficients		Standardized Coefficients	*t*	*p*	Durbin-Watson
		Beta	Std. Error	Beta			
Model 1	Constant	0.578	0.224		2.580	0.011	2.012
	FSS	0.290	0.074	0.326	3.916	<0.001	
R = 0.326 R^2^ = 0.106 Adjusted R^2^ = 0.099 F = 15.332							
Dependent Variable: DLQI							
Model 2	Constant	0.431	0.165		2.605	0.010	1.982
	ISS	0.499	0.079	0.484	6.283	<0.001	
R = 0.484 R^2^ = 0.234 Adjusted R^2^ = 0.228 F = 39.481							
Dependent Variable: DLQI							
Model 3	Constant	−0.304	0.240		−1.265	0.208	2.031
	FSS	0.262	0.065	0.294	4.026	<0.001	
	ISS	0.478	0.075	0.464	6.351	<0.001	
R = 0.566 R^2^ = 0.320 Adjusted R^2^ = 0.310 F = 30.170							
Dependent Variable: DLQI							

Abbreviations: FSS, Fear of Scabies Scale; DLQI, Dermatology Life Quality Index; ISS, Internalized Stigma Scale.

**Table 4:** Associations between the subdimensions of the internalized stigma scale and quality of life.

		Non-Standardized Coefficients		Standardized Coefficients	*t*	*p*	Durbin-Watson
		Beta	Std. Error	Beta			
Model 1	Constant	0.449	0.146		3.079	0.003	2.015
	Alienation	0.480	0.068	0.527	7.037	<0.001	
R = 0.527 R^2^ = 0.277 Adjusted R^2^ = 0.272 F = 49.515							
Dependent Variable: DLQI							
Model 2	Constant	0.995	0.155		6.435	<0.001	1.950
	Stereotype endorsement	0.228	0.076	0.255	2.991	0.003	
R = 0.255 R^2^ = 0.065 Adjusted R^2^ = 0.058 F = 8.948							
Dependent Variable: DLQI							
Model 3	Constant	0.632	0.108		5.867	<0.001	1.967
	Perceived discrimination	0.431	0.054	0.574	7.968	<0.001	
R = 0.574 R^2^ = 0.330 Adjusted R^2^ = 0.325 F = 63.493							
Dependent Variable: DLQI							
Model 4	Constant	0.381	0.123		3.102	0.002	2.136
	Social withdrawal	0.547	0.061	0.622	9.013	<0.001	
R = 0.622 R^2^ = 0.386 Adjusted R^2^ = 0.382 F = 81.233							
Dependent Variable: DLQI							
Model 5	Constant	1.390	0.211		6.574	<0.001	2.049
	Stigma resistance	0.021	0.088	0.021	0.236	0.813	
R = 0.021 R^2^ = 0.000 Adjusted R^2^ = −0.007 F = 0.056							
Dependent Variable: DLQI							

Abbreviations: DLQI, Dermatology Life Quality Index.

**Table 5:** Predictors of fear of scabies among patients with scabies.

Model	*β*	95% CI	*p*
Constant	-	2.040 to 2.967	0.001
Age	0.116	−0.006 to 0.016	0.413
Gender	−0.224	−0.475 to −0.066	0.010
Marital status	0.203	−0.044 to 0.515	0.098
Education level	0.468	0.202 to 0.583	<0.001
Place of residence	0.001	−0.477 to 0.482	0.991
Duration of symptoms	0.708	0.003 to 0.008	<0.001
Nocturnal pruritus	0.408	0.232 to 1.602	0.009
Skin lesions	0.263	0.057 to 0.235	0.002
Regional involvement	0.073	0.023 to 0.123	0.005

**Table 6:** Clinical predictors of DLQI in patients with scabies.

Model	*β*	95% CI	*p*
Constant	-	0.589 to 2.589	0.002
Duration of symptoms	0.206	−0.001 to 0.004	0.309
Nocturnal pruritus	−0.014	−0.830 to 0.775	0.945
Skin lesions	−0.127	−0.153 to 0.029	0.179
Response to treatment	−0.708	−1.060 to −0.587	<0.001

## Data Availability

In accordance with institutional ethical considerations and patient confidentiality requirements, the data are not publicly available. However, anonymized data can be made available from the corresponding author upon reasonable request for academic and scientific purposes.

## Abbreviations

The following abbreviations are used in this manuscript:

FSS	Fear of Scabies Scale
DLQI	Dermatology Life Quality Index
ISS	Internalized Stigma Scale
SD	Standard Deviation
SPSS	Statistical Package for the Social Sciences
WHO	World Health Organization

## References

[1] Engelman D., Yoshizumi J., Hay R.J., Osti M., Micali G., Norton S., Walton S., Boralevi F., Bernigaud C., Bowen A.C. (2021). A framework for scabies control. PLoS Neglected Trop. Dis..

[2] Heukelbach J., Feldmeier H. (2006). Scabies. Lancet.

[3] Romani L., Steer A.C., Whitfeld M.J., Kaldor J.M. (2015). Prevalence of scabies and impetigo worldwide: A systematic review. Lancet Infect. Dis..

[4] Delaš Aždajić M., Bešlić I., Gašić A., Ferara N., Pedić L., Lugović-Mihić L. (2022). Increased scabies incidence at the beginning of the 21st century: What do reports from Europe and the world show?. Life.

[5] World Health Organization (2023). Scabies. https://www.who.int/news-room/fact-sheets/detail/scabies.

[6] Spaziante M., Agresta A., D’Amato M., De Carli G., Tonziello G., Vantaggio V., Malatesta G.N., Girardi E., Barca A., Scognamiglio P. (2025). Post-COVID-19 resurgence of scabies’ cases in the Lazio Region, Italy: A new emerging public health threat?. Infect. Dis. Poverty.

[7] Redondo-Bravo L., Fernández-Martínez B., Gómez-Barroso D., Gherasim A., García-Gómez M., Benito A., Herrador Z. (2021). Scabies in Spain? A comprehensive epidemiological picture. PLoS ONE.

[8] Medina M., Mora N., Ramos F., Méndez-Boo L., Guiriguet C., Hermosilla E., Fàbregas M., Mas A., Rodoreda S., Coma E. (2022). Increase of Scabies Infestations During the COVID-19 Pandemic in Catalonia. medRxiv.

[9] Louka C., Logothetis E., Engelman D., Samiotaki-Logotheti E., Pournaras S., Stienstra Y. (2022). Scabies epidemiology in health care centers for refugees and asylum seekers in Greece. PLoS Neglected Trop. Dis..

[10] Karaca Ural Z., Çatak B., Ağaoğlu E.S.R.A. (2022). Prevalence of scabies in the COVID-19 pandemic period and determination of risk factors for scabies: A hospital-based cross-sectional study in Northeast Turkey. Acta Parasitol..

[11] Ayhan E. (2025). Status of the scabies outbreak before, during and after the COVID-19 pandemic: A single-center hospital-based retrospective evaluation. BMC Infect. Dis..

[12] Fernando D.D., Mounsey K.E., Bernigaud C., Surve N., Estrada Chávez G.E., Hay R.J., Currie B.J., Chosidow O., Fischer K. (2024). Scabies. Nat. Rev. Dis. Primers.

[13] Ugbomoiko U.S., Oyedeji S.A., Babamale O.A., Heukelbach J. (2018). Scabies in resource-poor communities in Nasarawa State, Nigeria: Epidemiology, clinical features and factors associated with infestation. Trop. Med. Infect. Dis..

[14] Yitageasu G., Asnake A.A., Gebrehana A.K., Kase B.F., Asebe H.A., Tigabie M., Demoze L. (2025). Burden of scabies in displacement settings: A systematic review and meta-analysis among forcibly displaced populations. PLoS Neglected Trop. Dis..

[15] Lopes M.J., da Silva E.T., Ca J., Gonçalves A., Rodrigues A., Mandjuba C., Nakutum J., D’Alessandro U., Achan J., Logan J. (2020). Perceptions, attitudes and practices towards scabies in communities on the Bijagós Islands, Guinea-Bissau. Trans. R. Soc. Trop. Med. Hyg..

[16] Koschorke M., Al-Haboubi Y.H., Tseng P.C., Semrau M., Eaton J. (2022). Mental health, stigma, and neglected tropical diseases: A review and systematic mapping of the evidence. Front. Trop. Dis..

[17] Hofstraat K., van Brakel W.H. (2016). Social stigma towards neglected tropical diseases: A systematic review. Int. Health.

[18] Dimitrov D., Szepietowski J.C. (2017). Stigmatization in dermatology with a special focus on psoriatic patients. Postep. Hig. Med. Dosw..

[19] Singg S. (2015). Scabies Awareness and Fear of Scabies Scale-10. J. Clin. Case Stu..

[20] Alharthi A.S., Alsofyani M.A., Alharthi W.K., Alsalmi S.A., Altalhi A.S., Alswat K.A. (2021). Assessment of knowledge and fear of scabies in a Saudi population. J. Multidiscip. Healthc..

[21] Yirgu R., Middleton J., Cassell J.A., Bremner S., Davey G., Fekadu A. (2024). Quality of life among adults with scabies: A community-based cross-sectional study in north-western Ethiopia. PLoS Neglected Trop. Dis..

[22] Worth C., Heukelbach J., Fengler G., Walter B., Liesenfeld O., Feldmeier H. (2012). Impaired quality of life in adults and children with scabies from an impoverished community in Brazil. Int. J. Dermatol..

[23] Tosun N., Tosun M., Dığış M., Younis M. (2025). Internalized stigma in acne vulgaris patients and relationship with quality of life, disease severity. Healthcare.

[24] Jin-Gang A., Sheng-Xiang X., Sheng-Bin X., Jun-Min W., Song-Mei G., Ying-Ying D., Jung-Hong M., Qing-Qiang X., Xiao-Peng W. (2010). Quality of life of patients with scabies. J. Eur. Acad. Dermatol. Venereol..

[25] Lake S.J., Engelman D., Sokana O., Nasi T., Boara D., Marks M., Whitfeld M.J., Romani L., Kaldor J.M., Steer A.C. (2022). Health-related quality of life impact of scabies in the Solomon Islands. Trans. R. Soc. Trop. Med. Hyg..

[26] He Z., Marrone G., Ou A., Liu H., Ma L., Huang Y., Li Y., Sun L., Bai Y., Liu W. (2020). Factors affecting health-related quality of life in patients with skin disease: Cross-sectional results from 8,789 patients with 16 skin diseases. Health Qual. Life Outcomes.

[27] Koç Yıldırım S., Öğüt N.D., Erbağcı E., Öğüt Ç. (2023). Scabies affects quality of life in correlation with depression and anxiety. Dermatol. Pract. Concept..

[28] Öztürkcan S., Ermertcan A.T., Eser E., Turhan Şahin M. (2006). Cross validation of the Turkish version of dermatology life quality index. Int. J. Dermatol..

[29] Badia X., Mascaró J.M., Lozano R., Cavide Research Group (1999). Measuring health-related quality of life in patients with mild to moderate eczema and psoriasis: Clinical validity, reliability and sensitivity to change of the DLQI. Br. J. Dermatol..

[30] Zachariae R., Zachariae C., Ibsen H., Mortensen J.T., Wulf H.C. (2000). Dermatology life quality index: Data from Danish inpatients and outpatients. Acta Derm. Venereol..

[31] Zachariae H., Zachariae R., Blomqvist K., Davidsson S., Molin L., Mørk C., Sigurgeirsson B. (2002). Quality of life and prevalence of arthritis reported by 5795 members of the Nordic Psoriasis Associations. Data from the Nordic Quality of Life Study. Acta Derm. Venereol..

[32] Sofuoğlu K., Atış T.H., Yıldız İ., Uraz M., Ertabaklar H., Okyay P. (2023). Uyuz Hastalığı Korkusu Ölçeğinin Türkçe Formunun Geçerlik Güvenirlik Çalışması. Halk Sağlığı Araştırma Uygulamaları Derg..

[33] Ritsher J.B., Otilingam P.G., Grajales M. (2003). Internalized stigma of mental illness: Psychometric properties of a new measure. Psychiatry Res..

[34] Ersoy M.A., Varan A. (2007). Reliability and Validity of the Turkish Version of the Internalized Stigma of Mental Illness Scale. Turk Psikiyatr. Derg..

[35] Firmin R.L., Lysaker P.H., McGrew J.H., Minor K.S., Luther L., Salyers M.P. (2017). The Stigma Resistance Scale: A multi-sample validation of a new instrument to assess mental illness stigma resistance. Psychiatry Res..

[36] Mitchell E., Wallace M.H., Marshall J., Whitfeld M., Romani L. (2024). Scabies: Current knowledge and future directions. Front. Trop. Dis..

[37] Engelman D., Cantey P.T., Marks M., Solomon A.W., Chang A.Y., Chosidow O., Enbiale W., Engels D., Hay R.J., Hendrickx D. (2019). The public health control of scabies: Priorities for research and action. Lancet.

[38] Paller A.S., Rangel S.M., Chamlin S.L., Hajek A., Phan S., Hogeling M., Castelo-Soccio L., Lara-Corrales I., Arkin L., Lawley L.P. (2024). Stigmatization and mental health impact of chronic pediatric skin disorders. JAMA Dermatol..

[39] Nalbant E.K., İmren I.G., Dölek G.T. (2024). Internalized stigma and its relationship with quality of life and perceived health status in rosacea and acne vulgaris: A comparative cross-sectional study. Cureus.

